# Virtual Screening Strategy and In Vitro Tests to Identify New Inhibitors of the Immunoproteasome

**DOI:** 10.3390/ijms241310504

**Published:** 2023-06-22

**Authors:** Giulia Culletta, Marco Tutone, Roberta Ettari, Ugo Perricone, Carla Di Chio, Anna Maria Almerico, Maria Zappalà

**Affiliations:** 1 Dipartimento di Scienze e Tecnologie Biologiche Chimiche e Farmaceutiche (STEBICEF), Università degli Studi di Palermo, Via Archirafi 32, 90123 Palermo, Italy; 2 Dipartimento di Scienze Chimiche, Biologiche, Farmaceutiche ed Ambientali, Università di Messina, Viale Annunziata, 98168 Messina, Italy; 3 Drug Discovery Unit, Fondazione Ri.MED, 90133 Palermo, Italy

**Keywords:** immunoproteasome, β1i subunit, β5i subunit, docking, induced fit docking, pharmacophore modeling, in vitro enzymatic assay

## Abstract

Immunoproteasome inhibition is a promising strategy for the treatment of hematological malignancies, autoimmune diseases, and inflammatory diseases. The design of non-covalent inhibitors of the immunoproteasome β1i/β5i catalytic subunits could be a novel approach to avoid the drawbacks of the known covalent inhibitors, such as toxicity due to off-target binding. In this work, we report the biological evaluation of thirty-four compounds selected from a commercially available collection. These hit compounds are the outcomes of a virtual screening strategy including a dynamic pharmacophore modeling approach onto the β1i subunit and a pharmacophore/docking approach onto the β5i subunit. The computational studies were first followed by in vitro enzymatic assays at 100 μM. Only compounds capable of inhibiting the enzymatic activity by more than 50% were characterized in detail using Tian continuous assays, determining the dissociation constant (*K*_i_) of the non-covalent complex where *K*_i_ is also the measure of the binding affinity. Seven out of thirty-four hits showed to inhibit β1i and/or β5i subunit. Compound **3** is the most active on the β1i subunit with *K*_i_ = 11.84 ± 1.63 µM, and compound **17** showed *K*_i_ = 12.50 ± 0.77 µM on the β5i subunit. Compound **2** showed inhibitory activity on both subunits (*K*_i_ = 12.53 ± 0.18 and *K*_i_ = 31.95 ± 0.81 on the β1i subunit and β5i subunit, respectively). The induced fit docking analysis revealed interactions with Thr1 and Phe31 of β1i subunit and that represent new key residues as reported in our previous work. Onto β5i subunit, it interacts with the key residues Thr1, Thr21, and Tyr169. This last hit compound identified represents an interesting starting point for further optimization of β1i/β5i dual inhibitors of the immunoproteasome.

## 1. Introduction

The ubiquitin–proteasome system (UPS) is the main non-lysosomal proteolytic system involved in the intracellular protein turnover in eukaryotic cells [1]. In normal cells, the proteasome proteolytic activity is responsible for regular cell cycle progression, homeostasis control, and immune surveillance [2,3]. Defects and aberrations in UPS can lead to uncontrolled cell proliferation and tumor development. For these reasons, novel approaches to cancer therapy exist based on proteasome inhibition [1,4,5].

The 20S constitutive proteasome is composed of four stacked rings: the two inner β-rings, which contain the catalytic subunit, and two outer α-rings, whose function is structural [6].

The catalytic subunits β1c, β2c, and β5c are responsible for the caspase-like (C-L), trypsin-like (T-L), and chymotrypsin-like (ChT-L) activities of the proteasome, respectively, with the ChT-L activity being currently considered the primary target for the development of novel anticancer agents [7].

In addition to the constitutive proteasome, vertebrates possess a specialized form of the proteasome, known as immunoproteasome, expressed in monocytes and lymphocytes and responsible for cell-mediated immunity and for the generation of major histocompatibility complex (MHC) class I ligands [8,9]. Under the stimuli of IFN-g and TNF-α, the βc core particles are replaced by β1i, β2i, and β5i subunits. While β2i and β5i perform the same type of activities of the β2c and β5c subunits, differently, the replacement of β1c with β1i brings the caspase-like activity to background levels. Prominent levels of immunoproteasome core particles have been detected in many autoimmune diseases, such as rheumatoid arthritis and systemic lupus erythematosus, and in a panel of inflammatory diseases, such as Crohn’s disease, inflammatory bowel disease, and ulcerative diseases. At the same time, overexpression of immunoproteasome core particles has been detected in hematological malignancies, such as multiple myeloma [9].

In this context, several β5i and/or β1i immunoproteasome-selective inhibitors have been identified [10,11,12]. Recently, particular attention has been devoted to the development of noncovalent proteasome inhibitors, which are free from side effects related to irreversible inhibition of a human target [13,14,15,16].

In this research area, our research group has been actively involved in the development of novel 20S proteasome inhibitors [17,18,19,20,21,22]. More recently, considering the variety of therapeutic applications of the development of immunoproteasome-selective inhibitors, we developed novel noncovalent immunoproteasome inhibitors.

**Scheme 1:**
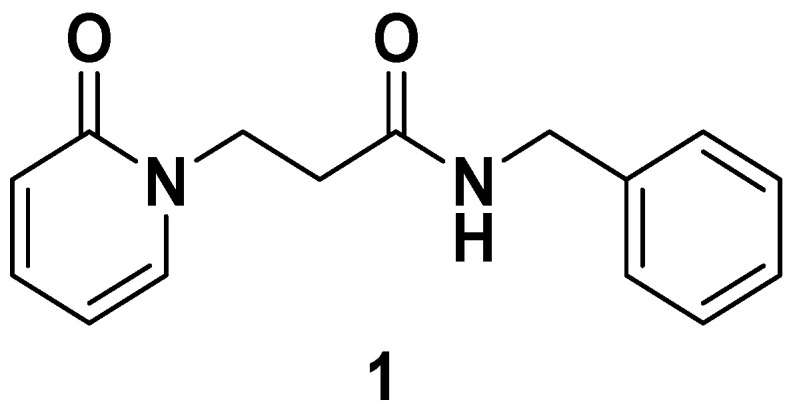
Structure of the selective β1i inhibitor **1**.

Among all the synthesized immunoproteasome inhibitors, we identified a panel of active inhibitors with *K*_i_ values in the low micromolar or sub-micromolar ranges towards the β5i and/or β1i subunits of immunoproteasome [23]. Compound **1**, *N*-benzyl-2-(2-oxopyridin-1(2*H*)-yl)propanamide (Scheme 1), was shown to be the most potent and selective inhibitor with a *K*_i_ value of 21 nM against the sole β1i subunit.

The mechanism of the non-covalent inhibition of this compound has been investigated using advanced molecular dynamics methods such as MD-binding (MDB) and Binding Pose MetaDynamics (BPMD). Moreover, an advanced docking method, Induced Fit Docking (IFD), was used for further investigation [24]. These approaches allowed for analyzing the binding mechanism, gaining insights into the ligand entrance pathway, and elucidating the dynamic behavior of the ligand within the binding cavity, providing a dynamic point of view for the definition of the pharmacophore features.

In this study, we employed these collected outcomes to obtain a dynamic pharmacophore model for β1i inhibitors. Additionally, to find new inhibitors of the β5i subunit, we developed a structure-based study using docking and pharmacophore models approaches, starting from the β5i subunit co-crystallized with PR-957 inhibitor (PDB ID: 3UNF).

Both pharmacophore models were used for a virtual screening campaign on commercially available compound collection of 2 million compounds (hereby called compound library) that allowed the selection of thirty-four potential inhibitors from three commercial databases for inhibition assays against β1i/β5i subunits.

## 2. Results and Discussion

### 2.1. β1i Pharmacophore Modeling

The previous MD study [24] allowed identifying three representative poses (pose 1, pose 2, and pose 3) that elucidated the dynamic behavior and stability of compound **1** within the binding cavity. The interactions pattern of inhibition included two H-bonds between the amide group and Ser21 and Gly47, a π-stacking between the benzyl group and Phe31, and a cation-pi stacking interaction between the 2-pyridone moiety and the epsilon amino group of Lys33. These pose models were used to carry out a pharmacophore approach for the β1i subunit.

**Figure 1:**
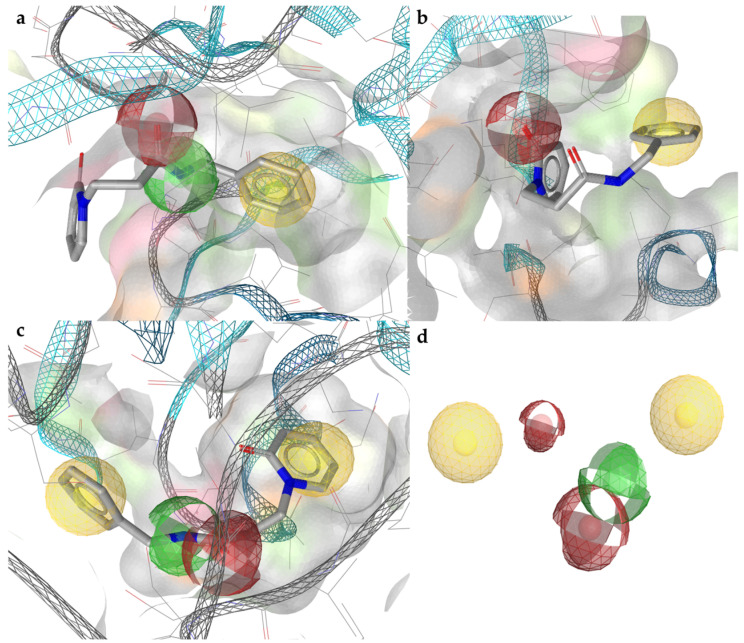
LigandScout pharmacophore model of (**a**) Pose1, (**b**) Pose2, (**c**) Pose3, and (**d**) Merged. Aromatic interactions are represented in yellow spheres, hydrogen bond donors in green spheres, and hydrogen bond acceptors in red spheres.

**Figure 2:**
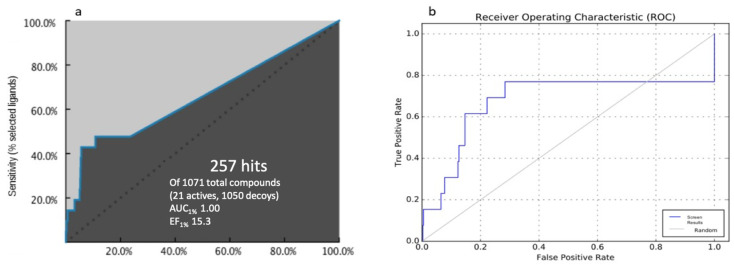
ROC curves of (**a**) LigandScout model and (**b**) PHASE model for β1i subunit.

For each pose, pharmacophore models were generated using LigandScout [25] PHASE [26]. In LigandScout, pose1 showed three features: a hydrogen bond acceptor (HBA), a hydrogen bond donor (HBD), and a hydrophobic interaction (Figure 1a). Pose2 showed a hydrogen bond acceptor (HBA) and a hydrophobic interaction (Figure 1b). Pose3 was characterized by one hydrogen bond acceptor (HBA), one hydrogen bond donor (HBD), and two hydrophobic interactions (Figure 1c). A merged pharmacophore model [27,28] including all features observed for each pose was constructed and improved by removing the redundant features. The final model is characterized by five features: two hydrogen bond acceptors (HBA), one hydrogen bond donor (HBD), and two hydrophobic interactions (Figure 1d). The model was retrospectively validated using the active and decoy datasets and performing the receiver operating characteristic (ROC) curve analysis. The ROC curves plot the sensitivity (true positive rate-TPR) against the specificity (false positive rate-FPR). The area under the curve (AUC) is used to assess the accuracy of the model. The grey dashed line represents the random line (AUC = 0.5); the blue curve represents the AUC of the model (AUC must be greater than 0.5). The enrichment factor is the number of active compounds found compared to their presence in the entire database. The model built with LigandScout showed values of AUC_1%_ = 1 and EF_1%_ = 15.3 (Figure 2a).

**Figure 3:**
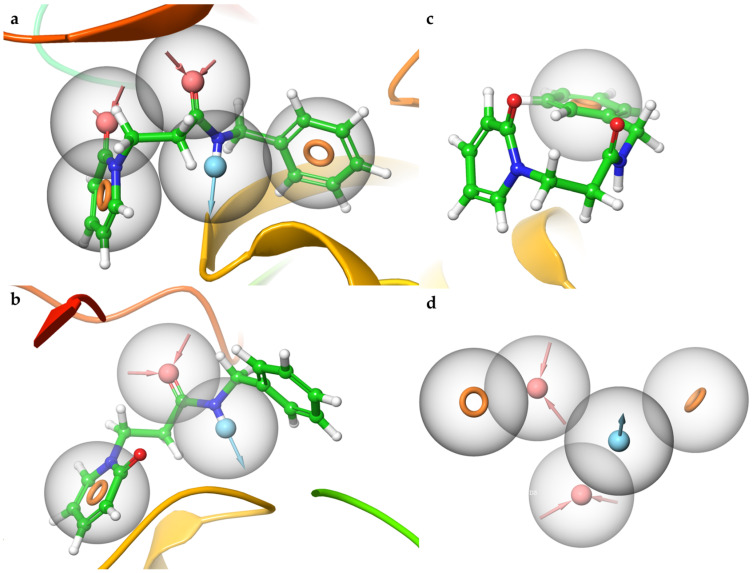
PHASE pharmacophore models of (**a**) Pose1, (**b**) Pose2, (**c**) Pose3, and (**d**) Merged. The aromatic rings are represented in orange rings, hydrogen bond donors in blue spheres, and hydrogen bond acceptors in pink spheres.

The same procedure was carried out with PHASE. Pose1 showed three features: two hydrogen bond acceptors, one hydrogen bond donor, and two aromatic rings (Figure 3a). In Pose2, one aromatic ring was recovered (Figure 3b). Pose3 was characterized by one hydrogen bond acceptor, one hydrogen bond donor, and one aromatic ring (Figure 3c).

As previously, the PHASE-merged pharmacophore model was then obtained with all features observed in the three poses. Two hydrogen bond acceptors, one hydrogen bond donor, and two aromatic rings (Figure 3d).

This model was also retrospectively validated and showed values of AUC_1%_ = 0.60 and EF_1%_ = 4.9 (Figure 2b).

### 2.2. β5i Docking and Pharmacophore Modeling

**Figure 4:**
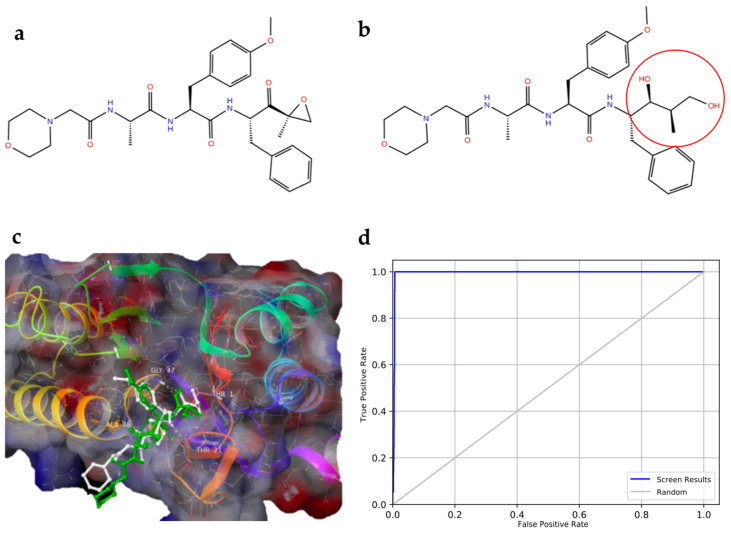
(**a**) PR-957 covalent epoxyketone; (**b**) Modified PR-957, free of the epoxy ring portion (circled in red); (**c**) Superposition of the re-docked poses of PR-597: modified (green) and co-crystallized (white); (**d**) ROC curve for the retrospective analysis of β5i subunit.

For the β5i subunit, a structure-based study was carried out on the experimental structure of the immunoproteasome (PDB ID: 3UNF). In this experimental structure of immunoproteasome, the β5i subunit is complexed with the PR-957 inhibitor. This ligand was covalently bound to Thr1 of the β5i subunit. The PR-957 inhibitor was structurally modified by breaking the covalent bond and filling in the open valence [29]. The portion of the epoxy ring responsible for the covalent attack on Thr1 was opened (Figure 4a,b).

A re-docking protocol was performed using Glide with XP precision to validate the ability to reproduce the experimental position of the co-crystallized ligand. The experimental and docking positions differ only in the epoxy moiety responsible for the covalent interactions. The root means square deviation (RMSD) between the re-docked and crystal conformation of PR-957 was 1.77 Å, less than 2 Å, indicating that Glide XP has a reliable scoring function for this study. The docking protocol was further validated using a β5i validation database of active and decoy compounds. The model showed values of AUC_1%_ = 0.90 and EF_1%_ = 27 (Figure 4c,d).

**Figure 5:**
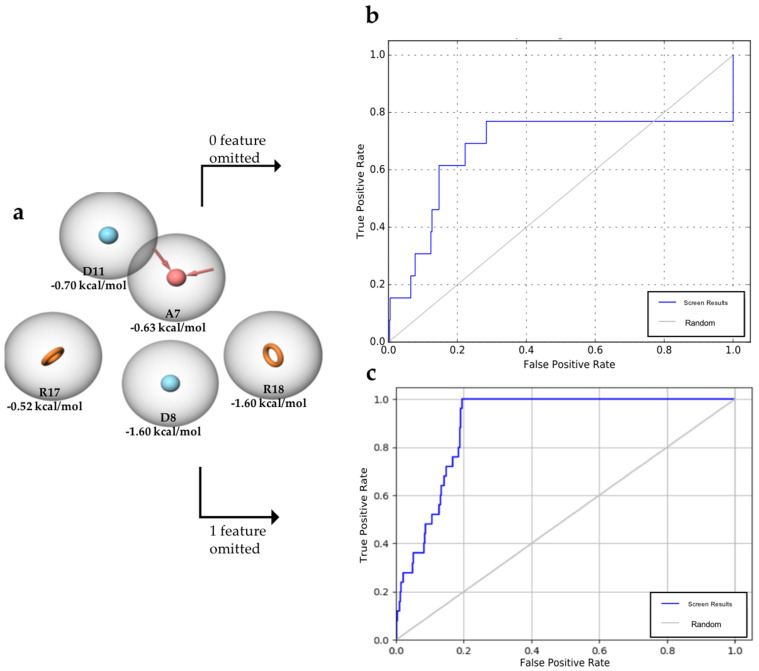
(**a**) E-pharmacophore hypothesis for β5i, five features: two aromatic rings (orange), two hydrogen bond donors (blue), and a hydrogen bond acceptor (red). (**b**) ROC curves, zero feature omitted and (**c**) one feature omitted.

Moreover, the docking method employed reports more than simple contact scoring by combining structural and energetic information between PR-957 and the active site. The docking post-processing outcomes were used to develop an e-pharmacophore hypothesis. The pharmacophore features with an energetic value < 0.5 kcal/mol were retained and used to compose the pharmacophore hypothesis. The e-pharmacophore hypothesis includes one hydrogen bond acceptor (A7), two hydrogen bond donors (D8 and D11), and two aromatic rings (R17 and R18). The energy values for the favorable features in the hypothesis are A3 −0.63 kcal/mol, D8 −1.60 kcal/mol, D11 −0.70 kcal/mol, R17 −0.52 kcal/mol, and R18 −1.60 kcal/mol (Figure 5a). The higher absolute value of the feature energy indicates that the ligand atom mapping has a higher interaction energy with amino acids. This five-point pharmacophore model was retrospectively validated. It showed AUC_1%_ = 0.40 and EF_1%_ = 2, so we decided to recalculate these values by omitting at least one feature. Figure 5b,c shows the ROC curves for the e-pharmacophore hypothesis with features zero and one omitted. The AUC_1%_ improved up to 0.84.

**Figure 6:**
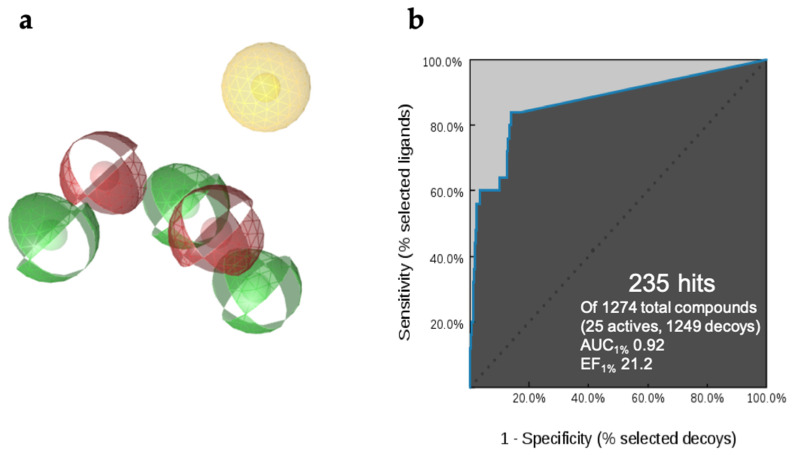
(**a**) LigandScout model for β5i, six features: an aromatic interaction (yellow), three hydrogen bond donors (green), and two hydrogen bond acceptors (red). (**b**) ROC curve.

LigandScout was also used to build a static pharmacophore starting from the docked position of PR-597. The model was improved by removing redundant features. Then, a six-feature model was obtained: three hydrogen bond acceptors (HBA), two hydrogen bond donors (HBD), and one aromatic interaction (Figure 6a) and a retrospective validation showed AUC_1%_ = 0.92 and EF_1%_ = 21.2 (Figure 6b).

### 2.3. β1i and β5i Virtual Screening

The pharmacophore models obtained from the β1i dynamic studies were used to perform a virtual screening on the compound library (VSβ1i).

The β5i pharmacophore models were used to perform the virtual screening campaign on the same compound library (VSβ5i) together with a docking analysis. The VSβ5i started with HTVS, SP, and XP docking screening. 1% of the prioritized ligands were retained and then submitted to pharmacophore matching.

The hits obtained from VSβ1i and VSβ5i were ranked according to the consensus score described in the Section 3. The consensus score improved the selection and retrieval of molecules shared between the pharmacophore models for β1 and β5. Finally, the hit molecules retrieved after consensus scoring were further evaluated with Induced Fit Docking (IFD) on β1i and β5i subunits. IFD confers more flexibility to the protein side chains, allowing the ligands to adjust and optimize binding interactions within the active sites. Visual inspection of the IFD results helped to enrich the final selection. Thirty-four molecules were selected and purchased for in vitro screening against β1i and β5i subunits using an enzymatic assay.

These hits were evaluated for their ability to inhibit both subunits of i20S by measuring the rate of hydrolysis of the corresponding fluorogenic substrate Ac-Pro-Ala-Leu-AMC for β1i and Suc-Leu-Leu-Val-Tyr-AMC for β5i. MG-132, a reversible inhibitor of the immunoproteasome, was used as a positive control for β1i and β5i.

Initially, compounds were screened against each proteolytic subunit at 100 μM. Only compounds capable of inhibiting the enzymatic activity by more than 50% were characterized in detail using Tian continuous assays. We determined the dissociation constant, i.e., *K*_i_, of the non-covalent complex [E▪I], where *K*_i_ is also the measure of the binding affinity, since the obtained linear progress curve exhibited a reversible, time-independent inhibition for all the active compounds.

To determine the *K*_i_ values (Table 1), seven different concentrations were selected for each compound that passed the initial screening, ranging from those that minimally inhibited to those that completely inhibited the activity of the immunoproteasome subunit.

The thirty-four identified hits are characterized by a central amide moiety except for compounds **17** and **31** which are, respectively, a purine derivative and a carboxylate derivative. Compounds **15**, **25**, and **35** have a central urea moiety. All the hits showed aromatic substituents on both sides of the amide moiety except for compounds **5**, **7**, **10**, **27**, and **29,** which have just aromatic substituents at one side of the central core. Compounds bearing pyrrole, pyrazole, and/or imidazole moieties showed no inhibition or low inhibition on β1i and β5i subunits, respectively.

**Figure 7:**
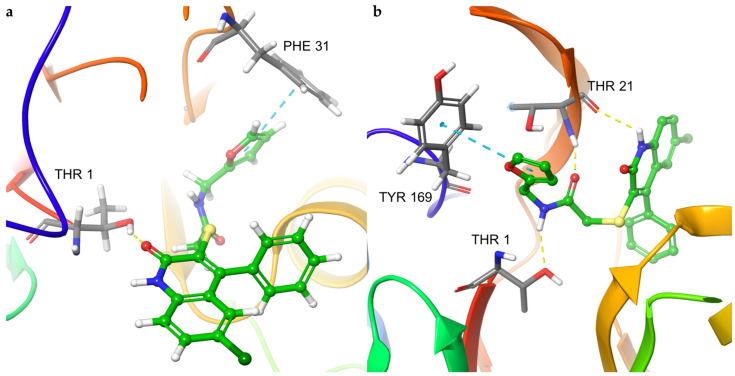
Binding mode of **2** into the active site of (**a**) β1i and (**b**) β5i subunits. The H-bonds are represented in yellow dashes and the π-stacking in blue dashes.

As shown in Table 1, some of the tested compounds were active on the immunoproteasome, with *K*_i_ values in the micromolar range (**2**, **3**, **13**, **14**, **17**, **30**, and **34**). Interestingly, as observed in the in silico studies, compound **2** inhibits both subunits (*K*_i_ = 12.53 ± 0.18 μM for β1i and 31.95 ± 0.81 μM for β5i). In Figure 7, the binding modes of compound **2**, obtained by IFD, are reported. The central amide moiety establishes an H-bond with the key residue Thr13 in both subunits. The furane ring forms a π-stacking interaction with Phe31 and Tyr169 in β1i and β5i subunits, respectively. An additional H-bond is established with Thr21 and the quinolinone moiety in β5i.

Compounds **3** and **30** inhibit β1i subunits. Structurally, compound **3** shows an isosteric substitution of an oxygen atom in place of a sulfur atom and a naphthyl ring in place of a quinolinone moiety. This moiety is replaced by a triazolopyrimidine in compound **30**, which is bound to the amide moiety without the thioether or ether chain as in the previous inhibitor. Compounds **13**, **14**, **17,** and **34** inhibit β5i. Interestingly, compound **17** is the only inhibitor that does not bear an amide moiety in the central core.

**Scheme 2:**
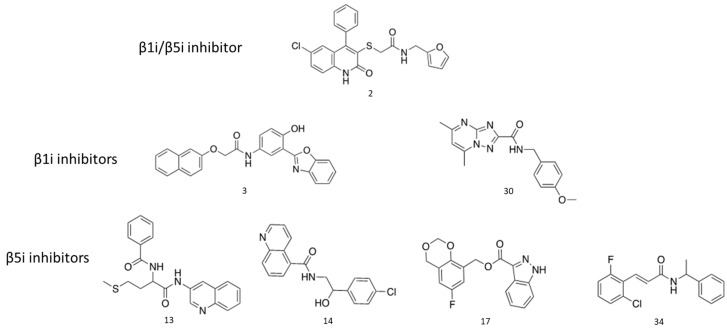
Most active compounds retrieved.

These newly identified hits could be interesting for further optimization. The amide moiety remains a key pharmacophore that is surrounded by the right combination of aromatic moieties and could lead to more active non-peptide and non-covalent inhibitors (Scheme 2).

## 3. Materials and Methods

### 3.1. System and Ligand Preparations

For this study, we selected the amino acid chain K from the 20S mouse immuno-proteasome crystallographic structure at 2.90 Å resolution (PDB ID: 3UNF) [30], corresponding to the β5i subunit with the co-crystallized ligand PR-957.

As reported in the previous paper, the reactive residue at the catalytic site, in this case, Thr1, was reconstituted after the removal of the covalent inhibitor by breaking the covalent bond and filling the open valence. Then, the structure and PR-957 were refined and optimized using the Protein Preparation Wizard (Schrödinger New York, 2021-1) and LigPrep (Schrödinger New York, 2021-1). OPLS2005 [31] was used as the force field and Epik (Schrödinger New York, 2021-1) was selected as the ionization tool at pH 7.2 ± 0.2.

A validation dataset containing active and decoy compounds was prepared for virtual screening. Active compounds on β1 and β5 subunits were obtained from the literature [23,32,33,34,35] for both subunits, while decoys were obtained from the DUD-E database [36] and filtered to remove duplicates. For each active compound in the dataset, 50 decoys were generated. An in-house and two commercial libraries (Asinex and Bioscient) containing >300,000 compounds were prepared in SDF format. The libraries were pre-filtered to remove groups classified as Tox-Alerts, Pan Assays INterference compoundS (PAINS), and Rapid Elimination Of Swills (REOS), which can give false positives due to non-specific reactions during testing [37,38]. As for PR-957, validation, in-house, and commercial libraries were prepared and optimized using LigPrep.

### 3.2. Pharmacophore Modeling Generation

LigandScout and PHASE (Schrödinger, LLC, New York, NY, USA, 2021) were used to generate dynamic pharmacophore models for β1i and structure-based pharmacophore model based on the experimental structure of the β5i subunit.

The models were generated using the following merged pharmacophore creation settings: Feature tolerance scale factor = 1.0; Maximum number of resulting pharmacophores = 10; Number of omitted features for merged pharmacophore = 4.

The structure-based pharmacophore model for the β5i subunit was generated, starting from the PDB coordinate set of β5i subunit-PR-957. The resulting models were validated for their performance in discriminating between the active and decoy molecules using specific databases for β1i and β5i.

A consensus score was calculated. It combined the normalized scores obtained from the Pharmacophore-Fit Score from LigandScout and the Phase-Fit Score from Phase, as shown in Equation (1).

(1) $$Consensus=\left(\frac{Pharmacophore\;Fit\;Score}{10}+\frac{Phase\;Score}{3}\right)$$

### 3.3. Molecular Docking

The docking study was performed using the Glide docking tool v. 9.0. [39].

The grid box was set on the PR-957 ligand coordinates. The Van der Waals radius was 1 Å, and the partial charge cut-off was 0.15 Å with flexible ligand sampling. Bias sampling torsion penalization for amides with non-planar conformation and Epik state penalties were added to the docking score. To validate the docking protocol and evaluate the ability to reproduce the experimental pose of the co-crystallized ligand, the re-docking test was performed with XP-level precision. The validation of the docking is carried out through the calculation of the RMSD of the cognate ligand, which gives an indication of the deviation between the experimental pose of the ligand and that reproduced by the docking algorithm. The reference value for RMSD is <2.0 for the scoring function.

The Virtual Screening was performed in three different docking steps: High Throughput Virtual Screening (HTVS), Standard Precision (SP), and Extra Precision (XP). Each docking step was run unconstrained.

### 3.4. Induced Fit Docking (IFD)

Induced Fit Docking (IFD) developed by Schrödinger [40] was performed using the standard protocol, as reported in the different studies [24,29,41,42,43,44,45]. The receptor box for β5i was set on the PR-957 pose with no constraints applied. The receptor box for β1i was defined on the residues Ser21, Phe31, Ser33, and Gly47.

### 3.5. In Vitro 20S Immunoproteasome Inhibition Assays

Human 20S immunoproteasome, obtained from the human spleen, was purchased from Enzo Life Science. The hydrolysis of the appropriate peptidyl 7-amino-4-methyl coumarin (AMC) substrate was monitored to measure the proteolytic activities of the immunoproteasome. The substrates Suc-Leu-Leu-Val-Tyr-AMC (Bachem, Bubendorf, Switzerland) for β5i and Ac-Pro-Ala-Leu-AMC (Biomol GmbH, Hamburg, Germany) for β1i subunits were used at 50 μM. Fluorescence of the hydrolysis product AMC was measured at 30 °C with a 380 nm excitation filter and a 460 nm emission filter using an Infinite 200 PRO microplate reader (Tecan, Männedorf, Switzerland). A preliminary screening at 100 μM inhibitor concentrations was carried out on the two proteolytic activities of the immunoproteasome.

An equivalent amount of DMSO as a negative control and compound **1** and MG-132 (a reversible immunoproteasome inhibitor) as a positive control were used for β1i and β5i, respectively. Compounds showing at least 50% inhibition at the screening concentration were then progressed into detailed assays. Continuous assays were performed at seven different concentrations ranging from 100 µM to 5 µM and by calculating the dissociation constants *K*_i_ of the enzyme–inhibitor complex using the Cheng–Prusoff equation, *K*_i_ = IC_50_/(1 + [S]*K*_m_^−1^). Inhibitor solutions were prepared from stocks in DMSO. Each independent assay was duplicated in 96-well plates with a total volume of 200 mL. For the assay on β1i and β5i subunits, a human 20S immunoproteasome was incubated at 30 °C at a final concentration of 4 μg/mL with the inhibitor at seven different concentrations. The reaction buffer comprised Tris·HCl (50 mM, pH 7.4), KCl (25 mM), NaCl (10 mM), MgCl_2_ (1 mM), and 0.03% SDS. AMC released from substrate hydrolysis was monitored in a kinetic cycle for 10 min.

## 4. Conclusions

In this work, we report a virtual screening strategy built on a dynamic pharmacophore modeling approach onto the β1i subunit and a pharmacophore/docking approach onto the β5i subunit of the immunoproteasome. The outcomes of the computational approaches led to the identification of thirty-four hit compounds selected from three libraries. These hit compounds were tested in in vitro enzymatic assays and seven (**2**, **3**, **13, 15**, **17**, **30**, and **34**) were active on the immunoproteasome, with *K*_i_ values in the micromolar range. Compound **3** and compound **17** are the most active against the β1i subunit and β5i subunit, respectively. Interestingly, Compound **2** showed inhibitory activity on both subunits, and induced fit docking analysis revealed a binding pattern onto β1i with Thr1 and Phe31 that represent new key residues as reported in our previous work. Onto β5i subunit, it interacts with the key residues Thr1, Thr21, and Tyr169. The hit compounds identified represent an interesting starting point for further optimization. Compound **2** showed a dual inhibition and some key pharmacophoric features that could be exploited to identify more potent inhibitors. Moreover, we are planning to investigate the anti-inflammatory profile of β5i-inhibitors and the anticancer activity of β1i-inhibitors, since an up-regulation of the corresponding βi subunits has been detected in inflammatory diseases or hematological malignancies, respectively, in future studies.

## Figures and Tables

**Table 1:** Inhibitory potencies: *K*_i_ values (μM) or percentage of inhibition at 100 μM of selected compounds on β1i and β5i subunits. n.i. is for no inhibition.

Compound	Structure	β1i	β5i
**2**	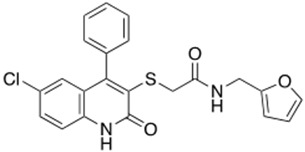 2-((6-chloro-2-oxo-4-phenyl-1,2-dihydroquinolin-3-yl)thio)-*N*-(furan-2-ylmethyl)acetamide	12.53 ± 0.18	31.95 ± 0.81
**3**	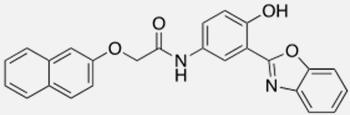 *N*-(3-(benzo[d]oxazol-2-yl)-4-hydroxyphenyl)-2-(naphthalen-2-yloxy)acetamide	11.84 ± 1.63	9%
**4**	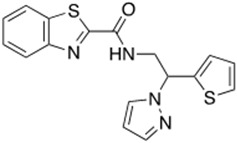 *N*-(2-(1*H*-pyrazol-1-yl)-2-(thiophen-2-yl)ethyl)benzo[d]thiazole-2-carboxamide	n.i.	23%
**5**	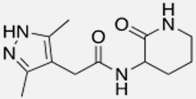 2-(3,5-dimethyl-1*H*-pyrazol-4-yl)-*N*-(2-oxopiperidin-3-yl)acetamide	n.i.	4%
**6**	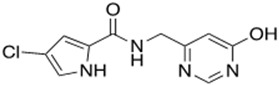 4-chloro-*N*-((6-hydroxypyrimidin-4-yl)methyl)-1*H*-pyrrole-2-carboxamide	n.i.	17%
**7**	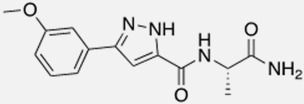 (*S*)-*N*-(1-amino-1-oxopropan-2-yl)-3-(3-methoxyphenyl)-1*H*-pyrazole-5-carboxamide	n.i.	18%
**8**	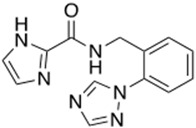 *N*-(2-(1H-1,2,4-triazol-1-yl)benzyl)-1*H*-imidazole-2-carboxamide	n.i.	18%
**9**	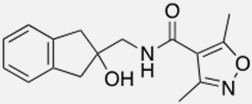 *N*-((2-hydroxy-2,3-dihydro-1*H*-inden-2-yl)methyl)-3,5-dimethylisoxazole-4-carboxamide	24%	10%
**10**	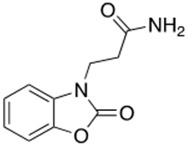 3-(2-oxobenzo[d]oxazol-3(2*H*)-yl)propanamide	n.i.	28%
**11**	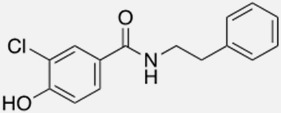 3-chloro-4-hydroxy-*N*-phenethylbenzamide	18%	10%
**12**	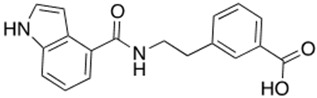 3-(2-(1*H*-indole-4-carboxamide)ethyl)benzoic acid	2%	23%
**13**	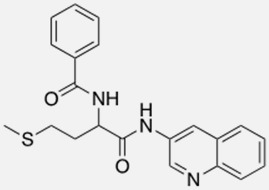 *N*-(4-(methylthio)-1-oxo-1-(quinolin-3-ylamino)butan-2-yl)benzamide	40%	43.68 ± 4.02
**14**	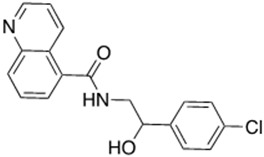 *N*-(2-(4-chlorophenyl)-2-hydroxyethyl)quinoline-5-carboxamide	47%	48.30 ± 4.97
**15**	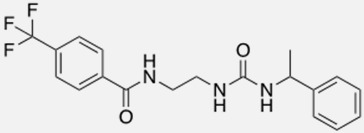 *N*-(2-(3-(1-phenylethyl)ureido)ethyl)-4-(trifluoromethyl)benzamide	24%	23%
**16**	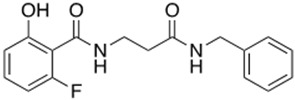 *N*-(3-(benzylamino)-3-oxopropyl)-2-fluoro-6-hydroxybenzamide	46%	22%
**17**	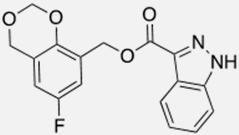 (6-fluoro-4*H*-benzo[d][1,3]dioxin-8-yl)methyl 1*H*-indazole-3-carboxylate	33%	12.50 ± 0.77
**18**	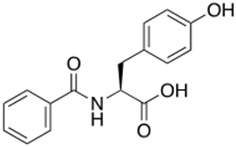 (*S*)-2-benzamido-3-(4-hydroxyphenyl)propanoic acid	15%	1%
**19**	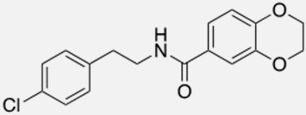 *N*-(4-chlorophenethyl)-2,3-dihydrobenzo[b][1,4]dioxine-6-carboxamide	3%	5%
**20**	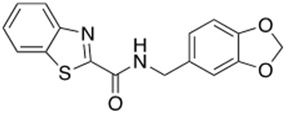 *N*-(benzo[d][1,3]dioxol-5-ylmethyl)benzo[d]thiazole-2-carboxamide	25%	36%
**21**	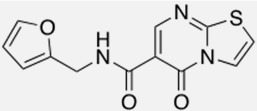 *N*-(furan-2-ylmethyl)-5-oxo-5*H*-thiazolo[3,2-a]pyrimidine-6-carboxamide	19%	23%
**22**	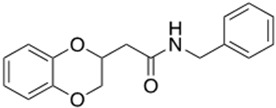 *N*-benzyl-2-(2,3-dihydrobenzo[b][1,4]dioxin-2-yl)acetamide	31%	32%
**23**	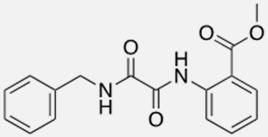 Methyl2-(2-(benzylamino)-2-oxoacetamido)benzoate	20%	34%
**24**	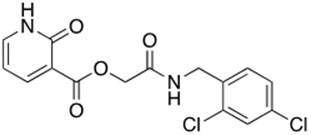 2-((2,4-dichlorobenzyl)amino)-2-oxoethyl 2-oxo-1,2-dihydropyridine-3-carboxylate	21%	34%
**25**	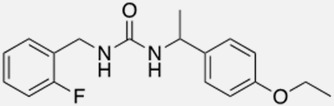 1-(1-(4-ethoxyphenyl)ethyl)-3-(2-fluorobenzyl)urea	16%	55%
**26**	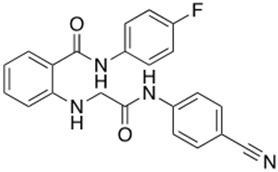 2-((2-((4-cyanophenyl)amino)-2-oxoethyl)amino)-*N*-(4-fluorophenyl)benzamide	Fluorescent	Fluorescent
**27**	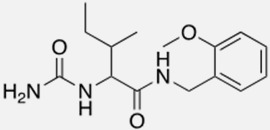 *N*-(2-methoxybenzyl)-3-methyl-2-ureidopentanamide	27%	23%
**28**	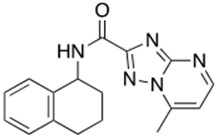 7-methyl-*N*-(1,2,3,4-tetrahydronaphthalen-1-yl)-[1,2,4]triazolo[1,5-a]pyrimidine-2-carboxamide	23%	13%
**29**	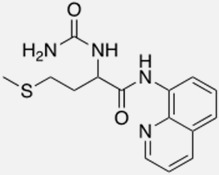 4-(methylthio)-*N*-(quinolin-8-yl)-2-ureidobutanamide	45%	47%
**30**	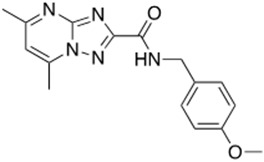 *N*-(4-methoxybenzyl)-5,7-dimethyl-[1,2,4]triazolo[1,5-a]pyrimidine-2-carboxamide	19.53 ± 2.83	23%
**31**	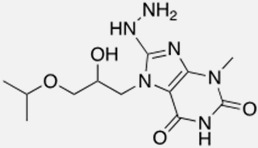 8-hydrazinyl-7-(2-hydroxy-3-isopropoxypropyl)-3-methyl-1*H*-purine-2,6(3*H*,7*H*)-dione	30%	37%
**32**	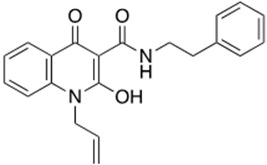 1-allyl-2-hydroxy-4-oxo-*N*-phenethyl-1,4-dihydroquinoline-3-carboxamide	32%	34%
**33**	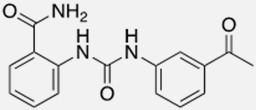 2-(3-(3-acetylphenyl)ureido)benzamide	17%	42%
**34**	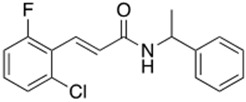 (*E*)-3-(2-chloro-6-fluorophenyl)-*N*-(1-phenylethyl)acrylamide	14%	48.13 ± 4.34
**MG-132**		0.13 ± 0.01	0.072 ± 0.014

## Data Availability

The authors will provide the data if requested.
